# Supplementary figures and images for: Comparative transcriptome analysis reveals key genes potentially related to organic acid and sugar accumulation in loquat
Source: PLoS One. 2021 Apr 29;16(4):e0238873. doi: 10.1371/journal.pone.0238873 (PMC8084190; doi:10.1371/journal.pone.0238873)

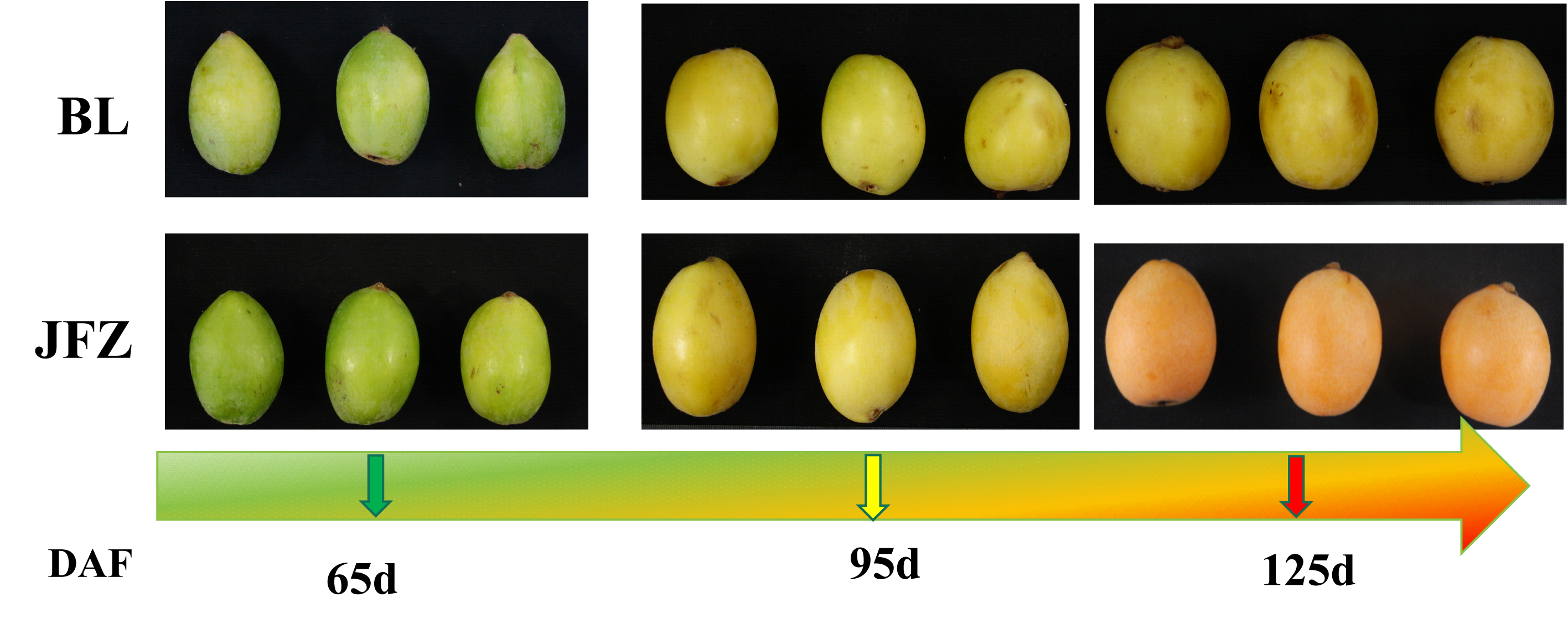

Supplement: S1 Fig — (TIF) [file pone.0238873.s001.tif]
