# Supplementary material for: Comparative transcriptome analysis reveals key genes potentially related to organic acid and sugar accumulation in loquat
Source: PLoS One. 2021 Apr 29;16(4):e0238873. doi: 10.1371/journal.pone.0238873 (PMC8084190; doi:10.1371/journal.pone.0238873)
Supplement: Attachment — Submitted filename: Response to Reviewers.docx [file pone.0238873.s010.docx]

Professor Xiaoming Pang

Editor, Plos one

July 8, 2020

Dear Dr. Xiaoming Pang

Thank you so much for critiquing our submission titled "Comparative transcriptome analysis reveals key genes potentially related to organic acid and sugar accumulation in loquat." We reviewed the concerns from the reviewers and have improved our manuscript. We have also utilized the service of an English language editing company to improve the language and presentation of our manuscript. Therefore, we have incorporated the suggested changes and would like to resubmit our manuscript.

We have incorporated the changes suggested by the reviewers and have answered their questions to the best of our ability. Please see our point-to-point responses below.

**Reviewer 1:**

1. In the abstract part, the author only described the results and lacks summative sentences.

We have added the summative sentence of the article from 2- Page 2, line 35–43 in the abstract part.

2. There is a lack of detailed description of the experimental materials, why these two experimental materials were used, why these three sampling times were chosen?

We selected the cultivars ‘JiFangZhong’ and ‘BaiLi’ because ‘JFZ’ has high acid and low sugar, while ‘BL’ has low acid and high sugar. Both cultivars are late maturing, and have similar flowering patterns. In addition, we also provide pictures of the three periods of two varieties in S1 Fig. Therefore, the differences in sugar/acid ratios in the two cultivars would provide a good overview of their accumulation and metabolism and the related genes. Chen et al, Hirai M and Yang (reference 6, 9 and 12 in the revised manuscript) have showed that these three time points are the critical period during the accumulation of louquat fruit sugar and acid. Thus we chose these three developmental stages in this study.

3. The genetic background of the two cultivars was somewhat different, so the credibility of DEGs was somewhat low.

A previous study by Chen et al. (reference 8 in the revised manuscript) have indicated that the two cultivars in this study all belong to *Eriobotrya japonica* Lindl. Thus, We think the two cultivars have a consistent genetic background.We have added *Eriobotrya japonica* Lindl to the title of the article.

4. There are only three sampling time points, and if there were more time points, the results would be more reliable.

First, according to a large number of article (reference 6, 9 and 12 in the revised manuscript), the three periods selected in this paper are the three key periods for the accumulation of sugar and acid in loquat fruit. Then, Jiang et (in Supplementary literature) have showed that the flesh of pummelo cv. GXMY and its mutants HRMY and HJMY in three development periods of fruit were selected to investigate the candidate genes of carotenoid metabolism by the RNA-Seq technique. Besides, Zhang et (in Supplementary literature) have also showed that seeds of P. rockii and P. lutea at 20, 60 and 80 days were collected to study FA biosynthesis metabolism by the RNA-Seq technique. . Thus We think the three points taken in this article can explain the accumulation of sugar and acid mechanism during the development of loquat fruit.

Format modification:

We have modified the format strictly in accordance with the format requirements of Plos one.

**Editor's Comments to Author:** Pay attention to the typos, for example, P43，polant families
Organic acids related taste was repeated several times in such a short introduction.The rationale of present study and the use of the two cultivars was not well presented.

We have modified the polant typo to plant, and have carefully checked other typos in the article. We have deleted some sentences about Organic acids related taste in introduction, such as P55 and P63. We have added some content about the rationale of present study and the use of the two cultivars in S1 Fig, P13, P68-72 and P84-87.if there are any further corrections necessary, we will be happy to incorporate them.

We look forward to your response. Thanks again for helping us improve this submission for your esteemed journal.

Best regards,
